# Dietary vitamin C intake and the risk of islet autoimmunity and type 1 diabetes: The Environmental Determinants of Diabetes in the Young (TEDDY) Study

**DOI:** 10.1007/s00394-026-04116-2

**Published:** 2026-09-24

**Authors:** Markus Mattila, Lazarus K. Mramba, Sari Niinistö, Iris Erlund, Xiang Liu, Ulla Uusitalo, Carin Andrén Aronsson, Sandra Hummel, Brigitte I. Frohnert, Hemang M. Parikh, Stephen S. Rich, William Hagopian, Jorma Toppari, Åke Lernmark, Anette G. Ziegler, Marian Rewers, Jeffrey P. Krischer, Leena Hakola, Jill M. Norris, Suvi M. Virtanen

**Affiliations:** 1 Faculty of Social Sciences, Unit of Health Sciences, Tampere University, Tampere, Finland; 2 Tampere University Hospital, Wellbeing Services County of Pirkanmaa, Tampere, Finland; 3 Department of Public Health, Finnish Institute for Health and Welfare, Helsinki, Finland; 4 Centre for Child, Adolescent and Maternal Health Research (TamCAM), Tampere University, Tampere, Finland; 5 Health Informatics Institute, Morsani College of Medicine, University of South Florida, Tampa, FL USA; 6 Finnish Institute for Health and Welfare, Helsinki, Finland; 7 Institute for Nutrition and Health Research, Helsinki, Finland; 8 Department of Clinical Sciences, Lund University Clinical Research Center, Skåne University Hospital, Malmö, Sweden; 9 Department of Pediatrics, Skåne University Hospital, Malmö, Sweden; 10 Institute of Diabetes Research, Helmholtz Zentrum München, German Research Center for Environmental Health, Munich-Neuherberg, Germany; 11 School of Medicine and Health, Technical University of Munich, Munich, and Forschergruppe Diabetes e.V., Neuherberg, Germany; 12 Barbara Davis Center for Childhood Diabetes, University of Colorado School of Medicine, Aurora, CO USA; 13 Department of Genome Sciences, University of Virginia, Charlottesville, VA USA; 14 Pacific Northwest Diabetes Research Institute, Seattle, WA USA; 15 Department of Pediatrics, Turku University Hospital, Turku, Finland; 16 Research Centre for Integrative Physiology and Pharmacology, Centre for Population Health Research, Institute of Biomedicine, and InFLAMES Research Flagship Centre, University of Turku, Turku, Finland; 17 Department of Epidemiology, Colorado School of Public Health, University of Colorado Denver, Aurora, CO USA

**Keywords:** Autoimmunity, Diet, Genotypes, Plasma ascorbic acid, Single nucleotide polymorphism, Type 1 diabetes, Vitamin C

## Abstract

**Purpose:**

We explored the associations between vitamin C intake and the risk of islet autoimmunity (IA) and/or type 1 diabetes in genetically at-risk children. Furthermore, we explored associations between vitamin C metabolism-related single nucleotide polymorphisms (SNPs) and type 1 diabetes outcomes.

**Methods:**

The current study within Environmental Determinants of Diabetes in the Young (TEDDY) cohort included 8478 children followed up every 3–6 months for relevant autoantibodies and diet. Dietary vitamin C intake was assessed longitudinally throughout childhood using age-specific dietary assessment methods and analysed using repeated measurements. Cox regression was used for the primary analyses and Bayesian joint longitudinal-survival models as sensitivity analyses.

**Results:**

A total of 777 (9.2%) children developed IA, including 292 (3.4%) with IAA-first and 337 (4.0%) with GADA-first autoimmunity; 319 (41.0%) progressed to type 1 diabetes. Mean (SD) vitamin C intake was 60.7 (38.8) mg/1000 kcal. Higher vitamin C intake was associated with an increased risk of IAA-first autoimmunity (adjusted hazard ratio 1.04; 95% confidence interval 1.00–1.07, per 10 mg/1000 kcal increase) while lowest and highest tertiles of intake were associated with increased risk of GADA-first autoimmunity as compared to mid-tertile [(1.65; 1.20–2.27) and (1.42; 1.02–1.98), respectively]. Sensitivity analyses using Bayesian joint longitudinal-survival models supported the primary findings and suggested nonlinear associations between vitamin C intake and the risks of IA and multiple IA. Associations between vitamin C-related SNPs and study outcomes did not withstand correction for multiple testing.

**Conclusion:**

Vitamin C intake was not consistently associated with IA or progression to type 1 diabetes. However, the observed nonlinear association, with lowest risk for moderate intakes, merits further investigation.

**Trial registration:**

NCT00279318, 06/09/2004.

**Supplementary Information:**

The online version contains supplementary material available at https://doi.org/10.1007/s00394-026-04116-2.

## Introduction

Type 1 diabetes may result from complex interaction between genes and environmental factors including diet [1–4]. Vitamin C (ascorbic acid, dehydroascorbic acid (DHA)) as a dietary antioxidant may play a role in preventing islet autoimmunity (IA) and development of type 1 diabetes [5, 6]. Vitamin C is an essential micronutrient required from diet as the human body cannot synthesize it endogenously [7]. Vitamin C absorption, tissue distribution, and excretion are tightly regulated and certain factors such as smoking, pregnancy, disease state, and genetic polymorphism alter the homeostasis [8]. In the previous Environmental Determinants of Diabetes in the Young (TEDDY) analysis, high plasma ascorbic acid concentration in childhood was associated with decreased risk of IA with insulin autoantibodies (IAA) as the first appearing autoantibody but not with IA with glutamic acid decarboxylase (GADA) as the first appearing autoantibody [9]. In a recent multiomics analysis, serum ascorbate concentrations was negatively associated with development of IA at an early age [10]. Previous studies assessing the association between vitamin C intake and type 1 diabetes risk were case-control studies [11–13]. Prospective findings are limited currently to maternal diet [14, 15]. Vitamin C transport proteins regulate vitamin C metabolism and tissue distribution and thus, mutations such as single nucleotide polymorphisms (SNPs) in the genes encoding these proteins might alter their function and further the association between vitamin C intake and plasma vitamin C concentrations. Lower plasma ascorbic acid concentrations has been observed in individuals with rs33972313 minor alleles as compared to those with major alleles in the sodium-dependent vitamin C transport protein *SLC23A1* gene [9, 16]. Furthermore, polymorphism in genes encoding DHA/glucose transporter, haptoglobin, glutathione S-transferase, and superoxide dismutase 2 have been associated with increased or decreased plasma ascorbic acid concentrations [17]. Whether these genetic variations influence the association between vitamin C intake and the development of IA and/or type 1 diabetes is not known. Furthermore, exposures might have a different effect on different type 1 diabetes endotypes, i.e., persons with different first appearing autoantibodies [18–20]. The aim of this study was to explore the association between dietary vitamin C intake and the risk of IA and progression from IA to type 1 diabetes in children genetically at risk for type 1 diabetes. We hypothesized that higher vitamin C intake is associated with decreased risk of IA and/or progression to type 1 diabetes. Secondly, we studied the association between vitamin C intake and the risk of IA relative to the first observed autoantibody, insulin or glutamic acid decarboxylase as well as IA with multiple autoantibodies. Thirdly, we studied whether SNPs in genes of vitamin C metabolism were associated with the development of IA and progression to type 1 diabetes, or if the SNPs modified the association between vitamin C intake and the development of IA and progression to type 1 diabetes. Finally, we explored whether SNPs in vitamin C metabolism modify the association between dietary intake of vitamin C and plasma ascorbic acid concentrations.

## Research design and methods

### Study population and design

The Environmental Determinants of the Diabetes in the Young (TEDDY) Study is a prospective cohort study aiming to identify environmental causes of type 1 diabetes in genetically at-risk children based on HLA haplogenotype. The study includes six clinical centers: three in the U.S. (Colorado, Georgia/Florida, and Washington) and three in Europe (Finland, Germany, and Sweden). Study design and methods have been previously published [21, 22]. Participating families or primary caretakers have provided a written informed consent for genetic screening and participation in prospective follow-up visits. The study is funded by the National Institutes of Health (NIH) and the National Institute of Diabetes and Digestive and Kidney Diseases (NIDDK) and approved by local Institutional Review Boards as well as monitored by an External Advisory Board.

A total of 424,788 newborn infants were screened for type 1 diabetes-associated HLA haplogenotypes between September 1, 2004, and February 28, 2010. Inclusion criteria were one of the HLA class II haplogenotypes; HLA-DR3/4, -DR4/4, -DR4/8, -DR3/3, and -DR4/4. Additional HLA-DR haplogenotypes -DR4/1, -DR4/13, -DR4/9, and -DR3/9 were included for children who had first-degree relatives (parent or sibling) with type 1 diabetes. Follow-up clinic visits took place at 3-month intervals until the age of 4 years, and every 6 months thereafter until 10 years of age or until the child is diagnosed with type 1 diabetes. Islet autoantibody positive participants were monitored every three months.

From the baseline data of 8,676 children, 8,478 were included in the current analysis (Supplementary Fig. 1). Persistent confirmed IA definition was the appearance of one or more islet cell autoantibodies: insulin (IAA), glutamic acid decarboxylase (GADA), or insulinoma antigen-2 (IA-2 A) confirmed at two or more consecutive visits. IA with multiple autoantibodies was defined as the appearance of more than one of the autoantibodies at two or more consecutive visits. Type 1 diabetes diagnosis was based on American Diabetes Association criteria [23]. During the follow-up, a total of 777 out of 8478 children (9.2%) developed IA. Of them, 292 (3.4%) children developed IA with IAA as the first appearing autoantibody (IAA-first), 337 (4.0%) children developed IA with GADA as the first appearing autoantibody (GADA-first), while 450 (5.3%) children developed IA with multiple autoantibodies (multiple IA). A total of 319 (41.0%) of the IA children progressed to type 1 diabetes. Outcome analyses in the current study included children up to 10 years of age. Information on the mode of delivery, birth order, maternal pre-pregnancy BMI, maternal education, and maternal smoking during pregnancy was received from questionnaires filled in by the parents during the first year of the study.

### Genotyping

For SNP genotyping, we used the ImmunoChip, an Illumina Infinium custom array which is based upon robust genome-wide association analyses (GWAS) in 12 autoimmune diseases, as well as the TEDDY-T1DExomeChip, a genome-wide array with exome content and additional custom (> 90,000) SNPs. A total of 69 common (minor allele frequency > 0.05) tagging SNPs were selected from regions containing genes associated with vitamin C metabolism; ascorbic acid transport (*SLC23A1*, *SLC23A2*), DHA/glucose transporter (*SLC2A1*, *SLC2A2*, *SLC2A4*) fructose transporter (*SLC2A5*), haptoglobin (*HP*), glutathione S-transferase (*GSTP1*), superoxide dismutase 2 (*SOD2*). The final analysis included 41 SNPs that passed quality control metrics and not in high (r2 > 0.9) linkage disequilibrium. We performed a genome-wide imputation analysis for the TEDDY cohort (*n* = 8,478) using Trans-Omics for Precision Medicine (TOPMed) freeze 8 reference panel, the 1000 Genomes, with the whole-genome sequencing data as reference panels to identify additional SNPs. Principal components analysis (PCA) using KING software was performed using each unrelated TEDDY participant to estimate ancestry, with the two most significant principal components used as covariates in statistical models.

### Dietary assessment

A 24-h recall was administered to the families at the initial TEDDY visit during the child’s age of 3 months. From 6 months onward, dietary intake was assessed using repeated 3-day food records collected every 3 months until 12 months of age and every 6 months thereafter. Participating families were instructed to fill in the food record during the subsequent 2 week and 1 weekend day/s at every designated age point. Any missing or unclear information was inquired by trained research nurses/nutritionists during every scheduled clinic visit. The dietary assessment used in TEDDY was described in detail before [24, 25]. Data on vitamin C in the food composition databases from all four countries were harmonized [26]. Information on dietary supplement use was collected prospectively during follow-up visits and nutrient intakes from supplements were calculated using TEDDY nutrient composition databases. Vitamin C intake from foods and dietary supplements were estimated separately and subsequently summed to derive total vitamin C intake.

### Plasma ascorbic acid measurements

Plasma samples measured for ascorbic acid were collected at the age of 6 months, 12 months, and then annually to 6 years of age, or until and including the time of autoantibody seroconversion or the visit just preceding the type 1 diabetes diagnosis. After sample collection at the clinical centers, 50 µL of sodium citrate plasma (BD Vacutainer^®^CPT™ Cell Preparation Tubes) was transferred into cryovials and 0.2 mL of 5% trichloroacetic acid and 200 mg disodium EDTA were added, with subsequent freezing at − 70 °C [27]. Ascorbic acid measurements were performed at the Finnish Institute for Health and Welfare (THL), Helsinki, Finland. Ascorbic acid was determined by an ion-paired, reversed-phase, high-performance liquid chromatographic method using electrochemical detection, as described [27]. Isoascorbic acid was used as an internal standard for the quantification of ascorbic acid.

### Statistical methods

Vitamin C intake at each follow-up interval was derived from the dietary assessment available at that specific visit and incorporated as a time-dependent exposure variable in the Cox regression analyses. Cause-specific Cox proportional hazard models with vitamin C intake as a time-dependent exposure were used to test the association with IAA-first, and GADA-first during the first 10 years of life. Participants were followed until they developed the outcome (first sample positive for autoantibodies), were censored at their last visit (last sample negative for autoantibodies), or at age of 10 years, which ever came first. Vitamin C intake was assessed up to the same point as follow-up on IA outcomes. The risk of progression from IA-to-type 1 diabetes analysis used dietary vitamin C intake assessed at the time of seroconversion as the exposure variable and modelled the time from seroconversion to type 1 diabetes diagnosis using Cox regression models. Vitamin C intake was adjusted for energy intake using the multivariable nutrient density method [28, 29] in which vitamin intake is divided by total energy intake and the energy is included as a covariate in the model. The interquartile range method was used to detect outliers, where (1,379/154,773) = 0.88% of vitamin C intake observations were identified as outliers and replaced with country- and age-specific median values using an IQR scale factor of 3, according to a previously published TEDDY methodology [30]. Vitamin C intake was first analyzed as a continuous variable. We assessed the linearity of associations by categorization (tertiles by country and visit) and with restricted cubic splines, for which, the linearity of the association was assessed via visual inspection for all outcomes. IA related outcome models were adjusted for HLA haplogenotype (DR3/4 [highest genetic risk] vs. no DR3/4), family history of type 1 diabetes (first-degree relative (FDR) vs. general population (GP)), sex (female vs. male), and country (the U.S., Finland, Germany, Sweden). The association of dietary vitamin C intake at seroconversion with the risk of progression from IA to type 1 diabetes was further adjusted for age at seroconversion. Loss to follow-up was considered a random event with no additional information provided in our database. Additionally, we performed sensitivity analyses, where vitamin C intake and the development of outcomes were assessed up to 6 years of age since we assessed the plasma ascorbic acid concentrations up to that age in our previous study [9].

The association between vitamin C metabolism related SNPs and the outcomes were modeled using Cox regression adjusted for HLA haplogenotype, family history of type 1 diabetes, sex, country, and the first two principal components to account for population stratification (ancestral heterogeneity). The continuous vitamin C intake interactions with SNPs, sex, FDR status, HLA haplogenotype, and country were explored by including interaction terms in the Cox regression model. All interactions between SNPs and dietary vitamin C intake were fitted separately in the models while adjusting for the other covariates given above. When corrected for multiple testing, two SNPs; rs11586615 in *SLC2A1* and rs5746109 in *SOD2* modified the association between vitamin C intake and multiple IA. However, there were only 1–2 children homozygous for the alternative alleles at these SNPs with the multiple IA outcome. Due to small numbers in these genotypes, these findings were underpowered and were not reported. In a separate analysis, we assessed whether the association between vitamin C intake and the outcomes changed during follow-up by including an interaction term between vitamin C intake and follow-up time in the Cox regression models.

A total of 3,051 plasma samples up to 6 years of age were collected from 1,525 children. The association between vitamin C intake and plasma ascorbic acid concentrations was analyzed using a generalized estimating equation (GEE) to consider the within-subject correlation in the longitudinal data. Continuous vitamin C intake interactions with SNPs, sex, FDR status, HLA haplogenotype, and country were analyzed. GEE models were adjusted for sex, FDR status, clinical visit, and country. The vitamin C interactions with SNPs were further adjusted for two principal components.

P-values for the SNP interaction and associations were corrected for multiple testing using the false discovery rate method.

As a sensitivity analysis, we fitted Bayesian joint longitudinal-survival models, which simultaneously model repeated vitamin C intake measurements and the time-to-event process. Unlike the primary time-dependent Cox models, joint models estimate participant-specific underlying vitamin C intake trajectories and account for within-person variability and measurement error. Models were fitted using both total vitamin C intake (foods and supplements combined) and vitamin C intake from foods only. The analyses were adjusted for the same covariates as the primary Cox regression models (HLA genotype, family history of type 1 diabetes, sex, and country). Analyses of progression from IA to type 1 diabetes were further adjusted for age at persistent autoantibody positivity and first autoantibody type. Additional sensitivity analyses were conducted using dietary assessments collected from ages 1 to 10 years. Posterior mean coefficients, 95% credible intervals (CrIs), and R-hat convergence diagnostics were examined for all models.

Data analysis was carried out using the Statistical Analysis System Software SAS^®^ Software 9.4 (SAS/STAT 15.3) [31] and R Core Team (2024) version 4.6.1 [32].

## Results

**Fig. 1:**
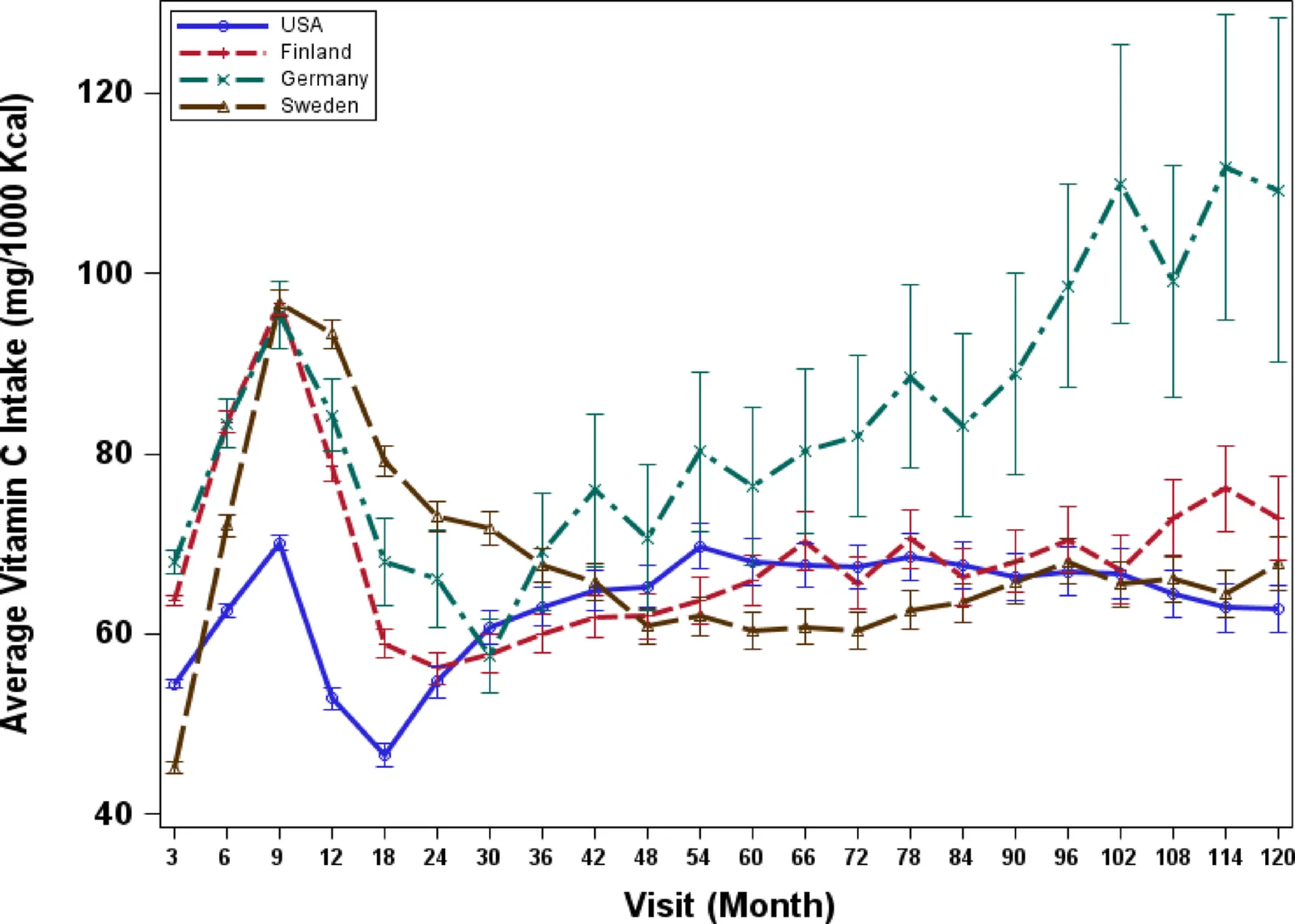
Mean energy adjusted total vitamin C intake by age and country

The distributions of background characteristics by outcomes are presented in Table 1. The mean total energy-adjusted vitamin C intake by country and age are presented in Fig. 1. Mean (SD) total vitamin C intake was 69.4 (54.8) mg/day and mean (SD) energy-adjusted vitamin C intake was 60.7 (38.8) mg/1000 kcal. Corresponding values for vitamin C intake from foods only were 65.3 (42.3) mg/day and 58.8 (38.2) mg/1000 kcal, respectively. Vitamin C supplement use and supplemental vitamin C intake by country during follow-up are presented in Table 2.

**Table 1:** Characteristics of TEDDY participants by background variables, n (%) unless otherwise indicated

Characteristic		No IA (*n* = 7,701)	IA (*n* = 777)	IAA-first (*n* = 292)	GADA-first (*n* = 337)	Multiple IA (*n* = 450)	Progression from IA to type 1 diabetes (*n* = 319)
Country	The U.S.	3,345	265 (34.1)	91 (31.2)	128 (38.0)	104 (32.6)	155 (34.4)
	Finland	1,606	194 (25.0)	93 (31.9)	67 (19.9)	95 (29.8)	118 (26.2)
	Germany	515	57 (7.3)	20 (6.8)	17(5.0)	31 (9.7)	38 (8.4)
	Sweden	2,235	261 (33.6)	88 (30.1)	125 (37.1)	89 (27.9)	139 (31.0)
Family history of type 1 diabetes	FDR: Yes	787	155 (20.0)	64 (21.9)	60 (17.8)	83 (26.0)	110 (24.4)
	FDR: No	6,914	622 (80.0)	228 (78.1)	277 (82.2)	236 (74.0)	340 (75.6)
Sex	Female	3,828	351 (45.2)	127 (43.5)	160 (47.5)	147 (46.1)	199 (44.2)
	Male	3,873	426 (54.8)	165 (56.5)	177 (52.5)	172 (53.9)	251 (55.8)
HLA haplogenotype	DR3/4: Yes	2,933	379 (48.8)	139 (47.6)	165 (49.0)	178 (55.8)	250 (55.6)
	DR3/4: No	4,768	398 (51.2)	153 (52.4)	172 (51.0)	141 (44.2)	200 (44.4)
Age at onset (months) median (IQR)		N/A	64.6 (35.0, 100.0)	22.2 (12.1, 45.9)	51.7 (27.2, 88.2)	75.2 (38.5, 111.5)	42.1 (16.8, 67.7)

**Table 2:** Vitamin C supplement use and supplemental vitamin C intake by country during follow-up

Country	*n* ^a^	Supplement use, *n* (%)	Supplement, mean (SD), mg/day
The US.	35,131	2823 (8.0)	47.1 (109.1)
Finland	18,499	1974 (10.7)	5.3 (18.9)
Germany	3,977	72 (1.8)	4.4 (39.5)
Sweden	31,323	1085 (3.5)	6.4 (54.0)
Total	88,930	5954 (6.7)	10.4 (53.8)

^a^ Values are presented at the observation level. Supplement use indicates visits at which vitamin C-containing supplements were reported

### Vitamin C intake and risk of IA and type 1 diabetes

The associations between vitamin C intake and the risk of IA and type 1 diabetes are presented in Table 3. In models with continuous exposure, the absolute (per 10 mg increase in intake) and energy-adjusted (per 10 mg/1000 kcal increase) total vitamin C intake was associated with increased risk of IAA-first but not with other outcomes.

**Table 3:** The risk of islet autoimmunity (IA) and progression from IA to type 1 diabetes associated with vitamin C intake

	IA cases *n* = 777		IAA-first cases *n* = 292		GADA-first cases *n* = 337		Multiple IA cases *n* = 450		Progression to type 1 diabetes cases *n* = 319	
	HR^a^ (95% CI)	*P*-value	HR^a^ (95% CI)	*P*-value	HR^a^ (95% CI)	*P*-value	HR^a^ (95% CI)	*P*-value	HR^b^ (95% CI)	*P*-value
Vitamin C intake										
+ 10 mg	1.00 (0.98-1.00)	0.694	**1.03 (1.00-1.07)**	**0.038**	0.98 (0.95–1.01)	0.185	1.01 (0.98–1.04)	0.450	0.97 (0.95–1.02)	0.454
T_1_	1.11 (0.90–1.36)	0.519	0.98 (0.70–1.36)	0.269	1.24 (0.91–1.70)	0.369	0.95 (0.73–1.25)	0.502	1.03 (0.76–1.39)	0.186
T_2_	1 (ref)		1 (ref)		1 (ref)		1 (ref)		1 (ref)	
T_3_	1.11 (0.91–1.36)		1.24 (0.91–1.68)		1.10 (0.80–1.50)		1.11 (0.86–1.43)		0.78 (0.58–1.07)	
+ 10 mg/1000 kcal^c^	1.00 (0.98–1.03)	0.733	**1.04 (1.00-1.07)**	**0.022**	0.98 (0.94–1.02)	0.300	1.02 (0.99–1.05)	0.089	0.99 (0.96–1.03)	0.880
T_1_^c^	1.24 (1.01–1.52)	0.103	1.06 (0.76–1.47)	0.340	**1.65 (1.20–2.27)**	**0.008**	0.92 (0.70–1.20)	0.746	0.83 (0.61–1.15)	0.363
T_2_^c^	1 (ref)		1 (ref)		1 (ref)		1 (ref)		1 (ref)	
T_3_^c^	1.18 (0.96–1.45)		1.25 (0.92–1.72)		**1.42 (1.02–1.98)**		1.01 (0.78–1.31)		0.82 (0.61–1.11)	

^a^ Adjusted for HLA haplogenotype, family history of type 1 diabetes, sex, and country

^b^ Adjusted for age at seroconversion, HLA haplogenotype, family history of type 1 diabetes, sex, and country

^c^ Further adjusted for energy by nutrient density method

Values in bold indicate statistically significant associations (P < 0.05)

**Fig. 2:**
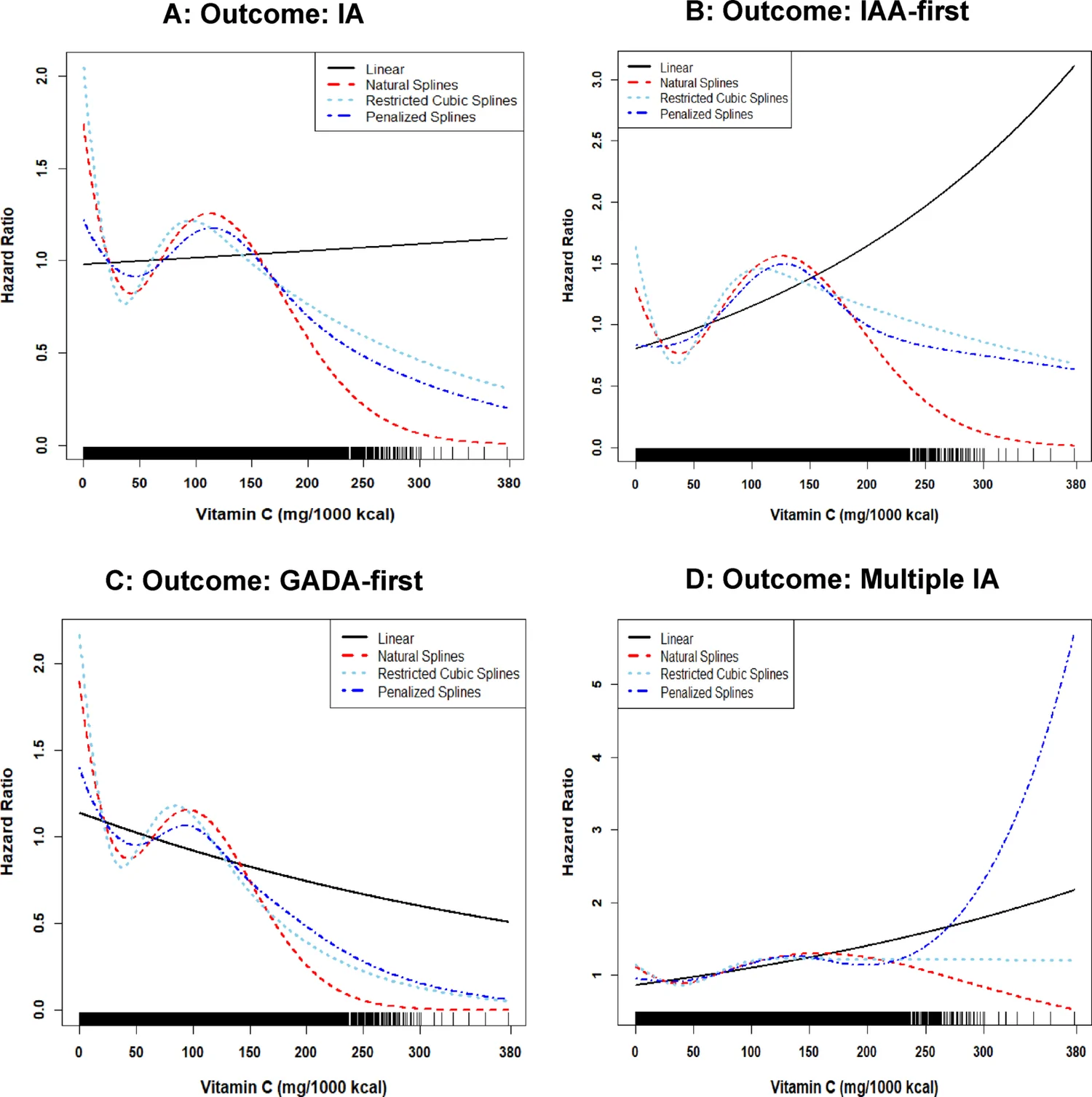
The shape of association between vitamin C intake and (**a**) Islet autoimmunity (IA), (**b**) IAA-first, (**c**) GADA-first, (**d**) multiple IA based on linear and splines

The categorization of exposure to tertiles showed an association between both high and low energy-adjusted vitamin C intake with increased risk of GADA-first (Table 3) suggesting non-linearity. The visual inspection for association between vitamin C and the risk of outcomes suggests a non-linear association between vitamin C intake and IA and IAA-first (Fig. 2, Supplementary Fig. 2). The number of observations with very high vitamin C intakes is low, which increases uncertainty when estimating the risk of IA at high intakes. The energy-adjusted intake of vitamin C was not associated with increased risk of outcomes in a sensitivity analysis in which the associations between vitamin C intake and the risk of outcomes were assessed up to 6 years of age (Supplementary Table 1). However, the lowest tertile of absolute intake of vitamin C was associated with increased risk of GADA-first.

FDR status, HLA haplogenotype, sex, or country did not modify the association between continuously modelled vitamin C intake and the risk of the outcomes. We did not observe an interaction over time between energy-adjusted vitamin C intake and the risk of outcomes, either for the full follow-up or the 6-year follow-up (Supplementary Table 2).

In sensitivity analyses using Bayesian joint longitudinal-survival models, evidence of nonlinear associations was observed between vitamin C intake and the development of IA and multiple IA (Supplementary Fig. 3, Supplementary Table 3). The results were largely consistent with the primary analyses (Table 3). Nonlinear associations were observed for IA and multiple IA, while positive linear associations were observed for IAA-first. Similar results were obtained when analyses were restricted to vitamin C intake from foods only and when dietary data collected before 1 year of age were excluded. No associations were observed for GADA-first autoimmunity or progression from islet autoimmunity to type 1 diabetes (Supplementary Fig. 3, Supplementary Table 3).

### Vitamin C metabolism-related SNPs and the risk of IA and type 1 diabetes

Several of the vitamin C metabolism-related SNPs were associated with the risk of IA and progression to type 1 diabetes when adjusted for HLA haplogenotype, family history of type 1 diabetes, sex, country, and ancestry (Supplementary Table 4). However, the associations were not significant when corrected for multiple testing.

### Vitamin C intake, SNPs, and plasma ascorbic acid concentration

Sex, FDR status, or HLA haplogenotype did not modify the association between continuously modeled vitamin C intake and plasma ascorbic acid concentrations, whereas country modified the association suggesting that the diet-plasma association is stronger in the U.S. and Finland in comparison to Germany and Sweden (Supplementary Table 5). Several SNPs modified the association between vitamin C intake and plasma ascorbic acid concentrations (Supplementary Table 6), although not withstanding multiple testing correction. The association between vitamin C intake and plasma ascorbic acid differed only slightly between genotypes.

## Discussion

In our large prospective birth cohort study, a high intake of vitamin C did not decrease the risk of IA or progression to type 1 diabetes. However, indications of a nonlinear association, with lowest risk for moderate intakes, were observed for IAA-first and GADA-first endpoints. SNPs in vitamin C metabolism genes might play a role in type 1 diabetes development, although the associations did not withstand multiple testing correction. Sensitivity analyses using Bayesian joint longitudinal-survival models produced findings broadly consistent with those of the primary time-dependent Cox regression analyses, supporting the robustness of the observed associations. Overall, these findings suggest a possible non-linear association between vitamin C intake and the risk of IA and multiple IA, particularly at the upper end of the intake distribution, whereas no evidence was observed for associations with GADA-first autoimmunity or progression to clinical type 1 diabetes.

### Vitamin C intake and the risk of IA and type 1 diabetes

We previously observed in TEDDY that high plasma ascorbic acid concentration were associated with decreased risk of IAA-first [9] and plasma vitamin C related metabolites [33]. Our current study used a different measure, dietary intake, and the results are less clear. Although we observed a direct association between vitamin C intake and IAA-first, the association was non-linear and thus should be interpreted with caution. For GADA-first both lowest and highest tertiles of vitamin C intake were directly associated with endpoint. These results suggest a non-linear association with moderate intake showing the lowest risk. However, the uncertainty related to high intakes due to low number of observations warrants caution with conclusions. The discrepancy between our previous and current observations on TEDDY could be explained by full cohort analysis in the current study vs. nested case-control design in our previous study. Furthermore, our previously observed inverse association between plasma ascorbic acid concentration and IAA-first suggested that vitamin C could protect from IA in early years of life [9]. Thus, we performed a sensitivity analysis where the association between vitamin C intake and the outcomes was limited to 6 years of age. We did not observe the same associations as in the main analysis for energy-adjusted intake of vitamin C. Instead, only the lowest tertile of absolute vitamin C intake was associated with increased risk of GADA-first, supporting the role of a beneficial effect for vitamin C. Furthermore, we did not observe time-dependent interaction between energy-adjusted intake of vitamin C and the outcomes which suggests that the associations did not change over time. The peak in vitamin C intake at approximately 9 months of age likely reflects the complementary feeding period, when vitamin C-rich fruits and infant foods are commonly introduced into the diet. The subsequent decline in energy-adjusted vitamin C intake may partly reflect increasing energy intake and dietary diversification during childhood. We also observed differences in intake trajectories between countries, particularly in Germany. Although the determinants of these differences were not examined in the present study, they may reflect country-specific dietary practices and food choices. To the best of our knowledge, there are no similar prospective studies exploring the association between vitamin C intake and the emergence of type 1 diabetes-specific autoantibodies. Results from previous retrospective case-control studies assessing the risk of type 1 diabetes are inconsistent. While an Australian study found that the use of vitamin C supplements was less frequent in children with type 1 diabetes before the onset than in control children [11], a Swedish study observed that higher vitamin C from diet was associated with an increased risk of type 1 diabetes although not when the association was stratified for nitrate and nitrite intakes [12]. A Canadian study found no association between vitamin C intake from food and the risk of type 1 diabetes [13]. Furthermore, no association has been observed between maternal vitamin C intake during pregnancy and the risk of IA or type 1 diabetes in cohort studies [14, 15].

### Vitamin C metabolism related genotypes and the risk of IA and type 1 diabetes

We observed that genotypes in ascorbic acid transporter *SLC23A2*, DHA/glucose transporter *SLC2A4*, fructose transporter *SLC2A5*, haptoglobin *HP*, glutathione S-transferase *GSTP1*, and *SOD2* were associated with increased or decreased risk of IA outcomes or progression to type 1 diabetes. However, none of the associations were significant after being corrected for multiple testing. Furthermore, we could not confirm our findings of previous nested case-control study where we observed an increased risk of type 1 diabetes in *SLC2A2* rs5400 minor genotype carriers [9]. This discrepancy may be attributed to differences in study design and sample size. It is also possible that the initial finding was a chance finding, or that the effect of rs5400 is modified by other genetic or environmental factors not accounted for in this cohort study. Genetic variants of these vitamin C metabolism related genes are suggested to alter the antioxidant defense and further disease development possibly via altered vitamin C homeostasis [17]. Thus, further studies are required to resolve controversies.

### Vitamin C metabolism related SNPs and plasma ascorbic acid concentrations

Higher vitamin C intake was associated with higher ascorbic acid concentrations, especially in the U.S. and Finland, compared to Germany and Sweden [34]. SNPs in *SLC23A2*, *SLC2A4*, *HP*, and *SOD2* modified the association between vitamin C intake and plasma ascorbic acid concentration. However, the differences between genotypes were small and the SNP interaction were not significant when corrected for multiple testing. In our previous biomarker study, we observed two additional genetic variants in RNA genes; *SNRPF-DT* rs117885456 and *LINC0122* rs56738967 that modified the association between vitamin C intake and plasma ascorbic acid concentration (37). In that study, SNPs related to dietary biomarkers were selected from the GWAS catalogue whereas in the current study, we selected SNPs from a much smaller number of specific genes known to be associated with vitamin C metabolism, although aware that other genetic variants could alter plasma ascorbic acid concentration and function.

### Strengths and limitations

The strength of our study is a large multinational study sample with consistent repeated highly standardized recording of diet and dietary supplement use during childhood, harmonized food composition databases, as well as regularly assessed plasma ascorbic acid concentration. Another strength is that our study included frequent and longitudinal follow-up of islet autoantibody assessments with identification of first-appearing islet autoantibodies which could represent different disease processes [35, 36]. Our genetic data included not only genotypes for ascorbic acid and DHA transporters, but also proteins regulating oxidative stress and detoxification of oxidized biomolecules as genetic variants of these proteins might affect plasma ascorbic acid concentrations [17]. Thus, we were able to study vitamin C metabolism related gene-nutrient interactions. Some limitations need to be acknowledged. Because vitamin C intake changes substantially during childhood and intake distributions differ between study countries, tertiles were derived separately by visit and country to facilitate comparisons among children at similar developmental stages. However, this approach limits the direct comparability of absolute intake levels across ages and countries and should be considered when interpreting the categorical analyses. Even that our food composition databases consider the loss of vitamin C during food processing, some loss may still occur due to vulnerability of vitamin C which could affect the accuracy of vitamin C intake assessment [37]. There is also a possibility of overreporting in the consumption of fruits and vegetables and other vitamin C rich foods for instance due to social approval bias [38] although we do not expect that the reporting would differ between those who developed the outcome and those who do not. Our study is observational, and therefore, we cannot conclude that our findings are causal. Children in our study have an increased risk of type 1 diabetes and thus our results may not apply to the general population. Although the analyses were adjusted for major established risk factors for IA and type 1 diabetes, residual confounding by other environmental determinants, such as maternal characteristics, infections, adiposity, or other dietary factors, cannot be excluded. Finally, our observations concerning specific SNPs and their associations and interactions with IA and type 1 diabetes development are novel and interesting, although they were not significant when corrected for multiple testing.

## Conclusions

In conclusion, our findings suggest no consistent relationship between vitamin C intake from food and dietary supplements and the risk of IA or type 1 diabetes.

## Supplementary Information

Below is the link to the electronic supplementary material.

Supplementary Material 1

## Data Availability

Data from The Environmental Determinants of Diabetes in the Young (https://doi.org/10.58020/y3jk-x087) reported here will be made available for request at the NIDDK Central Repository (NIDDK-CR) website, Resources for Research (R4R), https://repository.niddk.nih.gov/.

## Abbreviations

TEDDY: The Environmental Determinants of Diabetes in the Young Study
IAA: Insulin autoantibodies
GADA: Glutamic acid decarboxylase antibodies
IA-2A: Antibodies to tyrosine phosphatase-related islet antigen 2
DHA: Dehydroascorbic acid
SVCT: Sodium L-ascorbic acid transporter
SLC23A1: Solute Carrier Family 23 Member 1
SLC23A2: Solute Carrier Family 23 Member 2
SLC2A: Solute carrier family 2A
GLUT: Glucose transporter
HP: Haptoglobin
GSTP1: Glutathione S-transferase P1
SOD2: Superoxide dismutase 2
