# Supplementary material for: Dietary vitamin C intake and the risk of islet autoimmunity and type 1 diabetes: The Environmental Determinants of Diabetes in the Young (TEDDY) Study
Source: Eur J Nutr. 2026 Sep 24;65(7):262. doi: 10.1007/s00394-026-04116-2 (PMC13612646; doi:10.1007/s00394-026-04116-2)
Supplement: Supplementary file 1 [file 394_2026_4116_MOESM1_ESM.pdf]

European Journal of Nutrition

Dietary vitamin C intake and the risk of islet autoimmunity and type 1 diabetes: The Environmental Determinants of Diabetes in the Young (TEDDY) Study

Markus Mattila[1,2,3], Lazarus K. Mramba[4], Sari Niinistö[3], Iris Erlund[5], Xiang Liu[4], Ulla Uusitalo[4], Carin Andrén Aronsson[6,7], Sandra Hummel[8], Brigitte I. Frohnert[9] , Hemang M. Parikh, Stephen S. Rich[10], William Hagopian[11], Jorma Toppari[12,13], Åke Lernmark[6], Anette G. Ziegler[8], Marian Rewers[9], Jeffrey P. Krischer[4], Leena Hakola[1,2,3], Jill M. Norris[14], Suvi M. Virtanen[1,2,3,15] for the TEDDY Study Group

Author Affiliations:

[1]Faculty of Social Sciences, Unit of Health Sciences, Tampere University, Tampere, Finland

[2]Tampere University Hospital, Wellbeing Services County of Pirkanmaa, Tampere, Finland

[3]Department of Public Health, Finnish Institute for Health and Welfare, Helsinki, Finland

[4]Health Informatics Institute, Morsani College of Medicine, University of South Florida, Tampa, FL, United States

[5]Finnish Institute for Health and Welfare, Helsinki, Finland, and Institute for Nutrition and Health Research, Helsinki, Finland

[6]Department of Clinical Sciences, Lund University Clinical Research Center, Skåne University Hospital, Malmö, Sweden

[7]Department of Pediatrics, Skåne University Hospital, Malmö, Sweden

[8]Institute of Diabetes Research, Helmholtz Zentrum München, German Research Center for Environmental Health, Munich-Neuherberg, Germany, and Forschergruppe Diabetes at Klinikum rechts der Isar, School of Medicine and Health, Technical University of Munich, Munich, and Forschergruppe Diabetes e.V., Neuherberg, Germany

[9]Barbara Davis Center for Childhood Diabetes, University of Colorado School of Medicine, Aurora, CO, United States

[10]Department of Genome Sciences, University of Virginia, Charlottesville, VA, United States

[11]Pacific Northwest Diabetes Research Institute, Seattle, WA, United States

[12]Department of Pediatrics, Turku University Hospital, Turku, Finland

[13]Research Centre for Integrative Physiology and Pharmacology, and Centre for Population Health Research, Institute of Biomedicine, and InFLAMES Research Flagship Centre, University of Turku, Turku, Finland

[14]Department of Epidemiology, Colorado School of Public Health, University of Colorado Denver, Aurora, CO, United States
[15]Centre for Child, Adolescent and Maternal Health Research, Tampere University and Tampere University Hospital, Tampere, Finland

Corresponding Author: Markus Mattila, PhD, e-mail: markus.mattila@tuni.fi,

Supplementary Fig. 1 Participant flow chart

Supplementary Fig. 2 The shape of association between vitamin C intake and a) Islet autoimmunity (IA), b) IAA-first, c) GADA-first, d) multiple IA based on linear and restricted cubic splines (RC-splines) showing confidence intervals and distribution of vitamin C intake among users

Supplementary Table 1 The risk of type 1 diabetes related outcomes associated with vitamin C intake followed up to 6 years of age

Supplementary Table 2 Interaction by time between vitamin C intake and the outcomes by months and years

Supplementary Table 3 The association between vitamin C metabolism related SNPs and the risk of islet autoimmunity (IA) outcomes and progression from IA to type 1 diabetes outcomes

Supplementary Table 4 Interaction between background variables and vitamin C intake on plasma ascorbic acid concentrations

Supplementary Table 5 Interaction between vitamin C metabolism related genes and energy adjusted vitamin C intake on plasma ascorbic acid concentrations

Supplementary material: The TEDDY Study Group

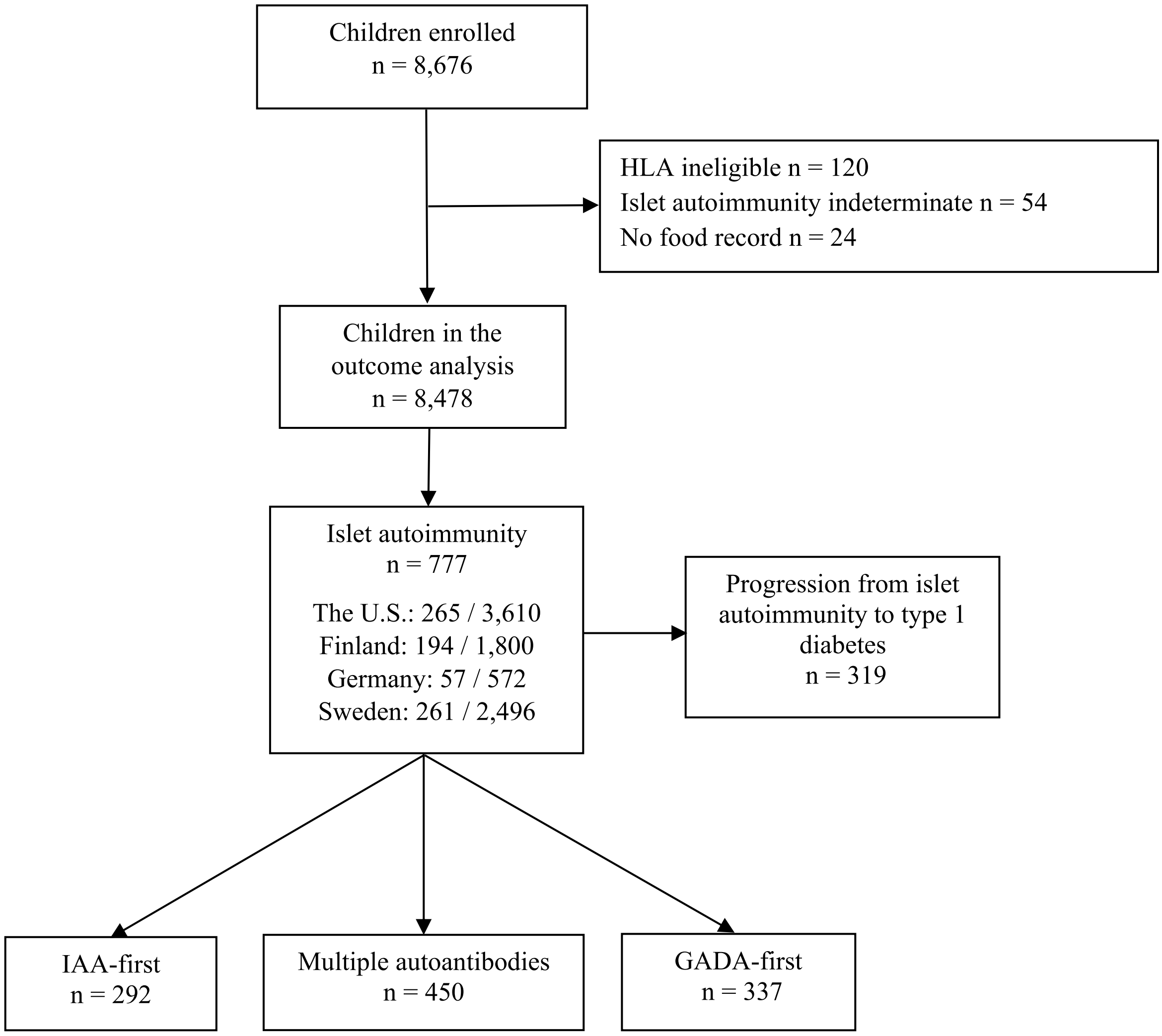

Supplementary Fig. 1 Participant flow chart

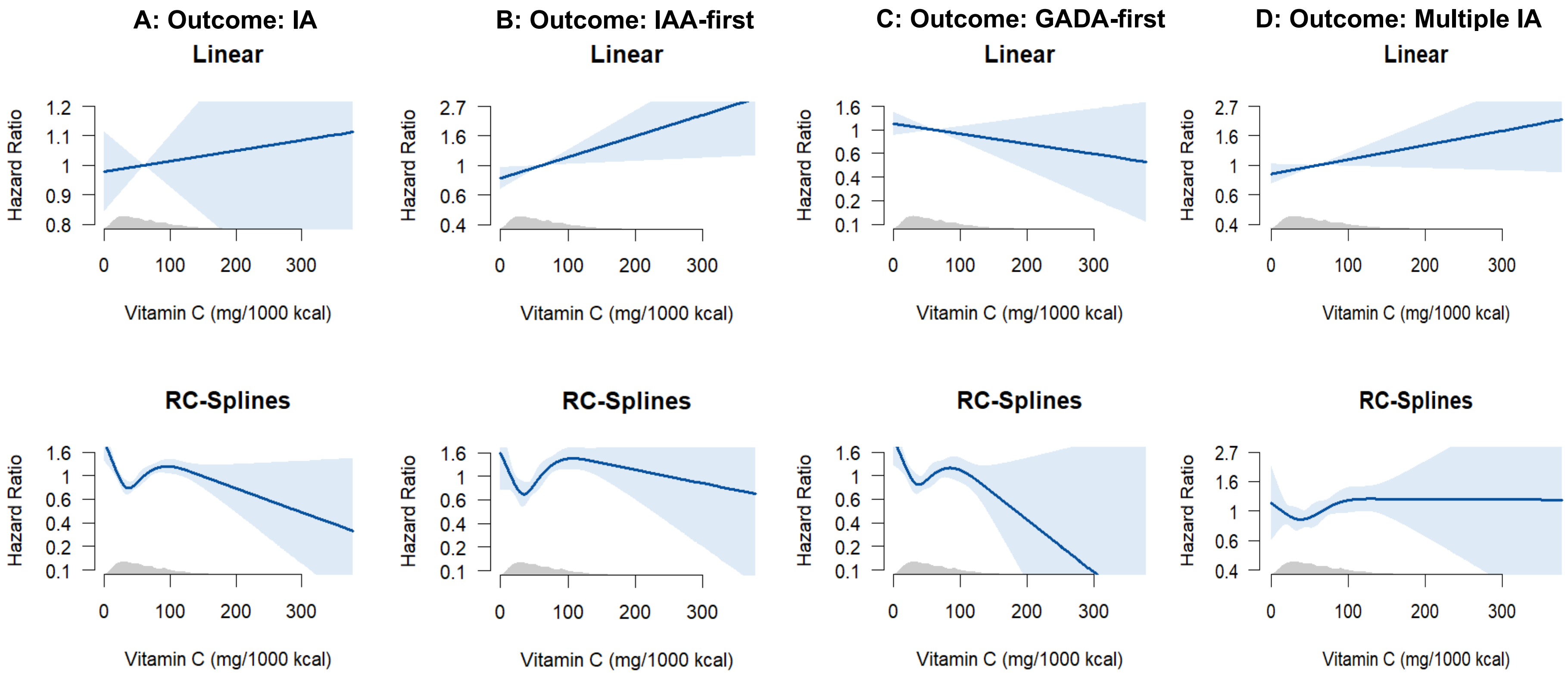

Supplementary Fig. 2 The shape of association between vitamin C intake and a) Islet autoimmunity (IA), b) IAA-first, c) GADA-first, d) multiple IA based on linear and restricted cubic splines (RC-splines) showing confidence intervals and distribution of vitamin C intake among users

Supplementary Table 1 The risk of type 1 diabetes related outcomes associated with vitamin C intake followed up to 6 years of age

	**IA**		**IAA-first**		**GADA-first**		**Multiple IA**		**Progression to type 1 diabetes**	
	**HR[a] (95% CI)**	**P-value**	**HR[a] (95% CI)**	**P-value**	**HR[a] (95% CI)**	**P-value**	**HR[a] (95% CI)**	**P-value**	**HR[b] (95% CI)**	**P-value**
Vitamin C intake										
+10 mg	1.00 (0.97-1.02)	0.782	1.03 (1.00-1.07)	0.077	0.97 (0.93-1.01)	0.122	1.02 (0.99-1.05)	0.206	0.99 (0.95-1.03)	0.749
T_1	1.13 (0.90-1.42)	0.496	1.04 (0.74-1.48)	0.463	**1.65 (1.14-2.38)**	**0.028**	0.98 (0.72-1.32)	0.277	1.02 (0.74-1.39)	0.378
T_2	1 (ref)		1 (ref)		1 (ref)		1 (ref)		1 (ref)	
T_3	1.12 (0.89-1.40)		1.22 (0.88-1.69)		1.39 (0.95-2.05)		1.20 (0.91-1.60)		0.82 (0.60-1.13)	
+10 mg/1000 kcal[c]	1.00 (0.98-1.03)	0.653	1.03 (1.00-1.06)	0.056	0.98 (0.93-1.02)	0.324	1.03 (1.00-1.06)	0.049	1.00 (0.96-1.04)	0.973
T_1[c]	1.19 (0.95-1.49)	0.285	1.07 (0.76-1.52)	0.575	1.36 (0.95-1.95)	0.231	0.94 (0.70-1.26)	0.491	0.76 (0.54-1.05)	0.194
T_2[c]	1 (ref)		1 (ref)		1 (ref)		1 (ref)		1 (ref)	
T_3[c]	1.15 (0.91-1.44)		1.19 (0.86-1.66)		1.13 (0.78-1.64)		1.12 (0.84-1.48)		0.81 (0.60-1.10)	

[a] Adjusted for HLA haplogenotype, family history of type 1 diabetes, sex, and country.

[b] Adjusted for age at seroconversion, HLA haplogenotype, family history of type 1 diabetes, sex, and country.

[c] Further adjusted for energy by nutrient density method

Supplementary Table 2 Interaction by time between vitamin C intake and the outcomes by months and years

	IA		IAA-first		GADA-first		Multiple IA		Progression to type 1 diabetes	
	Visit, months	Visit, years	Visit, months	Visit, years	Visit, months	Visit, years	Visit, months	Visit, years	Visit, months	Visit, years
	**P-value**	**P-value**	**P-value**	**P-value**	**P-value**	**P-value**	**P-value**	**P-value**	**P-value**	**P-value**
**Follow-up to 10 years**										
Absolute intake, median	0.75	0.93	0.75	0.51	0.03	0.03	0.20	0.17	0.04	0.03
Energy-adjusted intake, median	0.91	0.37	0.99	0.34	0.17	0.27	0.32	0.15	0.07	0.10
**Follow-up to 6 years**										
Absolute intake, median	0.34	0.69	0.59	0.40	0.01	0.01	0.99	0.76	0.39	0.19
Energy-adjusted intake, median	0.38	0.90	0.70	0.34	0.08	0.16	0.88	0.53	0.59	0.53

Supplementary Table 3 The association between vitamin C metabolism related SNPs and the risk of islet autoimmunity (IA) outcomes and progression from IA to type 1 diabetes outcomes

		**IA cases *n*=777**		**IAA-first cases *n*=292**		**GADA-first cases *n*=337**		**Multiple IA cases *n*=450**		**Progression to type 1 diabetes cases *n*=319**	
**Gene SNP (minor allele)**	**Genotype (% of children by genotype)**	**HR[a] (95% CI)**	**P-value**	**HR[a] (95% CI)**	**P-value**	**HR[a] (95% CI)**	**P-value**	**HR[a] (95% CI)**	**P-value**	**HR[b] (95% CI)**	**P-value**
SLC23A2 rs1715379 (A)	GG (25.47)					1 (ref)	**0.024**				
	GA (50.11)					1.08 (0.81-1.45)					
	AA (24.43)					**0.66 (0.45-0.99)**					
SLC2A4 rs5412 (A)	GG (68.12)									1 (ref)	**0.020**
	GA (28.27)									**0.69 (0.51-0.93)**	
	AA (3.61)									0.54 (0.27-1.08)	
SLC2A5 rs3004245 (C)	AA (31.58)					1 (ref)	**0.017**				
	AC (48.64)					**0.68 (0.51-0.90)**					
	CC (19.78)					0.94 (0.67-1.31)					
GSTP1 rs79512139 (A)	GG (83.46)									1 (ref)	**0.043**
	GA (15.91)									**1.43 (1.03-2.00)**	
	AA (0.63)									3.04 (0.73-12.65)	
HP rs8062041 (G)	AA (12.98)			1 (ref)	**0.019**						
	AG (46.47)			0.73 (0.51-1.03)							
	GG (40.55)			**0.59 (0.41-0.85)**							
HP rs5467 (A)	GG (63.80)									1 (ref)	**0.037**
	GA (32.15)									1.34 (0.99-1.80)	
	AA (4.05)									**1.79 (1.02-3.14)**	
SOD2 rs11752345 (A)	GG (58.76)							1 (ref)	**0.025**		
	GA (35.90)							**1.31 (1.04-1.65)**			
	AA (5.35)							**1.55 (1.00-2.41)**			

[a] Adjusted for HLA haplogenotype, family history of type 1 diabetes, sex, country, and the first two principal components.

[b] Adjusted for age at seroconversion, HLA haplogenotype, family history of type 1 diabetes, sex, country, and the first two principal components.

Supplementary Table 4 Interaction between background variables and vitamin C intake on plasma ascorbic acid concentrations

Background variables	P-value for interaction[a]	Association between vitamin C intake and plasma ascorbic acid concentrations	
		**GEE Estimate (SE)**	**P-value**
Country	**<0.001**		
The U.S.		0.0409 (0.0046)	**<0.001**
Finland		0.0351 (0.0028)	**<0.001**
Germany		0.0118 (0.0056)	**0.034**
Sweden		0.0137 (0.0031)	**<0.001**

[a] Adjusted for energy adjusted vitamin C intake, energy intake, sex, HLA haplogenotype, family history of type 1 diabetes, and country.

Supplementary Table 5 Interaction between vitamin C metabolism related genes and energy adjusted vitamin C intake on plasma ascorbic acid concentrations

Gene SNPs	Genotype (% of children by genotype)	P-value for interaction[a]	Association between vitamin C intake and plasma ascorbic acid concentrations	
			**GEE Estimate (SE)**	**P-value**
SLC23A2 rs6037988	CC (33.24)	**0.041**	0.0236 (0.0036)	<0.001
	CT (49.40)		0.0307 (0.0028)	<0.001
	TT (17.36)		0.0279 (0.0043)	<0.001
SLC2A1 rs3820546	GG (21.51)	**0.034**	0.0239 (0.0044)	<0.001
	GA (47.29)		0.0272 (0.0027)	<0.001
	AA (31.20)		0.0327 (0.0037)	<0.001
SLC2A4 rs5415	TT (48.06)	**0.019**	0.0328 (0.0027)	<0.001
	TA (41.72)		0.0265 (0.0030)	<0.001
	AA (10.21)		0.0156 (0.0060)	0.010
SOD2 rs56340441	AA (86.18)	**0.037**	0.0299 (0.0021)	<0.001
	AG (13.15)		0.0198 (0.0051)	0.001
	GG (0.67)		-0.0010 (0.0128)	0.939

[a]Adjusted for dietary vitamin C intake, energy intake, sex, HLA haplogenotype, family history of type 1 diabetes, country, and the first two principal components.

Supplementary material: The TEDDY Study Group

Colorado Clinical Center: Marian Rewers, M.D., Ph.D., PI[1,4,6,9,10], Kimberly Bautista[11], Judith Baxter[8,911], Daniel Felipe-Morales, Brigitte I. Frohnert, M.D., Ph.D.[2,13], Marisa Stahl, M.D.[12], Patricia Gesualdo[2,6,11,13], Michelle Hoffman[11,12,13], Randi Johnson, Ph.D.[2,3], Rachel Karban[11], Edwin Liu, M.D.[12], Jill Norris, Ph.D.[2,3,11], Holly O'Donnell, Ph.D.[8], Andrea Steck, M.D.[3,13], Kathleen Waugh[6,7,11]. University of Colorado, Anschutz Medical Campus, Barbara Davis Center for Childhood Diabetes, Aurora, CO, USA.

Finland Clinical Center: Jorma Toppari, M.D., Ph.D., PI[¥^1,4,10,13], Olli G. Simell, M.D., Ph.D., Annika Adamsson, Ph.D.[^11], Suvi Ahonen[*±§], Mari Åkerlund[*±§], Sirpa Anttila[µ¤], Leena Hakola, Ph.D.[*±], Sanni Heikura[µ¤], Tiia Honkanen[µ¤], Heikki Hyöty, M.D., Ph.D.[*±6], Jorma Ilonen, M.D., Ph.D.[¥3], Saori Itoshima, M.D.[¥^], Sanna Jokipuu[^], Taru Karjalainen[µ¤], Leena Karlsson[^], Jukka Kero, M.D., Ph.D.[¥^3, 13], Marika Korpela[µ¤], Jaakko J. Koskenniemi M.D., Ph.D.[¥^], Miia Kähönen[µ¤11,13], Mikael Knip, M.D., Ph.D.[*±], Minna-Liisa Koivikko[µ¤], Katja Kokkonen[*±], Merja Koskinen[*±], Mirva Koreasalo[*±§2], Kalle Kurppa, M.D., Ph.D.[*±12], Salla Kuusela, M.D. [µ¤], Jutta Laiho, Ph.D.[*6], Tiina Latva-aho[µ¤], Laura Leppänen[^], Katri Lindfors, Ph.D.[*12], Maria Lönnrot, M.D., Ph.D.[*±6], Elina Mäntymäki[^], Markus Mattila, Ph.D.[*±2], Maija E. Miettinen, Ph.D.[§2], Teija Mykkänen[µ¤], Tiina Niininen[±*11], Sari Niinistö, Ph.D.[§2], Noora Nurminen[*±], Sami Oikarinen, Ph.D.[*±6], Hanna-Leena Oinas[*±], Paula Ollikainen[µ¤], Zhian Othmani[¥], Sirpa Pohjola [µ¤], Jenna Rautanen[§], Mia Rein[µ¤], Minna Romo[^], Juulia Rönkä[µ¤], Nelli Rönkä[µ¤], Noora Ruotsalainen[µ¤], Satu Simell, M.D., Ph.D.[¥12], Päivi Tossavainen, M.D.[µ¤], Mari Vähä-Mäkilä[¥], Eeva Varjonen[^11], Riitta Veijola, M.D., Ph.D.[µ¤13], Irene Viinikangas[µ¤], Suvi M. Virtanen, M.D., Ph.D.[*±§2]. [¥]University of Turku, Turku, Finland, *Tampere University, Tampere, Finland, [µ]University of Oulu, Oulu, Finland, [^]Turku University Hospital, Wellbeing Services County of Southwest Finland, Turku, Finland, [±]Tampere University Hospital, Wellbeing Services County of Pirkanmaa, Tampere, Finland, [¤]Oulu University Hospital, Wellbeing Services County of North Ostrobothia, Oulu, Finland, [§]Finnish Institute for Health and Welfare, Helsinki, Finland.

Georgia/Florida Clinical Center: Richard McIndoe, Ph.D., PI[^4,10], Desmond Schatz*, M.D.[*4,7,8], Diane Hopkins[^11], Michael Haller, M.D.[*13], Melissa Gardiner[^11], Ashok Sharma[^], Ph.D.[^], Laura Jacobsen, M.D.[*13], Percy Gordon[^], Jennifer Hosford*,. [^]Center for Biotechnology and Genomic Medicine, Augusta University, Augusta, GA, USA. *University of Florida, Pediatric Endocrinology, Gainesville, FL, USA.

Germany Clinical Center: Anette G. Ziegler, M.D., PI[1,3,4,10], Ezio Bonifacio Ph.D.*, Cigdem Sanverdi, Anja Heublein, Sandra Hummel, Ph.D.[2], Annette Knopff[7], Melanie Köger, Sibylle Koletzko, M.D.[¶12], Claudia Ramminger[11], Roswith Roth, Ph.D.[8], Jennifer Schmidt, Marlon Scholz, Joanna Stock[8,11,13], Katharina Warncke, M.D.[13], Lorena Müller, Christiane Winkler, Ph.D.[2,11]. Forschergruppe Diabetes e.V. and Institute of Diabetes Research, Helmholtz Zentrum München, Forschergruppe Diabetes, and Klinikum rechts der Isar, Technische Universität München, Neuherberg, Germany. *Center for Regenerative Therapies, TU Dresden, Dresden, Germany, [¶]Dr. von Hauner Children's Hospital, Department of Gastroenterology, Ludwig Maximillians University Munich, Munich, Germany.

Sweden Clinical Center: Åke Lernmark, Ph.D., PI[1,3,4,5,6,8,9,10], Daniel Agardh, M.D., Ph.D.[6,12], Carin Andrén Aronsson, Ph.D.[2,11,12], Rasmus Bennet, Corrado Cilio, Ph.D., M.D.[6], Susanne Dahlberg, Malin Goldman Tsubarah, Emelie Ericson-Hallström, Lina Fransson, Emina Halilovic, Susanne Hyberg, Berglind Jonsdottir, M.D., Ph.D.[11], Naghmeh Karimi, Helena Elding Larsson, M.D., Ph.D.[6,13], Markus Lundgren, M.D., Ph.D.[13], Jessica Melin, Ph.D.[11], Kobra Rahmati, Anita Ramelius, Falastin Salami, Ph.D., Anette Sjöberg, Evelyn Tekum Amboh, Carina Törn, Ph.D.[3], Terese Wiktorsson. Lund University, Lund, Sweden.

Past staff: Eva Andersson, Marie Andersson Turpeinen, Rawya Antar, Maria Ask, Jenny Bremer, Sylvia Bianconi Svensson, Ulla-Marie Carlsson, Magdalena Delikat Kulinski, Annika Fors, Ulla Fält, Thomas Gard, Joanna Gerardsson, Monika Hansen, Anna Hansson, Carina Hansson, Gertie Hansson, Elin M. Hård af Segerstad, Ph.D.[2], Hanna Jisser, Fredrik Johansen, Linda Jonsson, Silvija Jovic, Sigrid Lenrick Forss, Barbro Lernmark, Ph.D.[8], Marielle Lindström, *Maria Markan, Theodosia Massadakis, Marlena Maziarz, Ph.D., Zeliha Mestan, Maria Månsson Martinez, Caroline Nilsson, Emma Nilsson, Yohanna Nordh, Karin Ottosson, Sara Rang, Anna Rosenquist, Monika Sedig Järvirova, Sara Sibthorpe, Birgitta Sjöberg, Ulrika Swartling Ph.D.[8], Erika Trulsson,* Ulrika Ulvenhag, *Anne Wallin, Ingrid Wigheden,* Åsa Wimar, *Sofie Åberg.*

Washington Clinical Center: William A. Hagopian, M.D., Ph.D., PI^[1,3,4,6,7,10,12,13], Michael Killian*[6,7,11,12], Claire Cowen Crouch*[11,13], Jennifer Skidmore*[2], Ben Kim*, Cody McCall*, Arlene Meyer*, Jared Radtke*, Shreya Roy*. ^Indiana University, Indianapolis, IN, USA. *Pacific Northwest Research Institute, Seattle, WA, USA.

Pennsylvania Satellite Center: Dorothy Becker, M.D., Margaret Franciscus, MaryEllen Dalmagro-Elias Smith[2], Ashi Daftary, M.D., Mary Beth Klein, Chrystal Yates. Children's Hospital of Pittsburgh of UPMC, Pittsburgh, PA, USA.

Data Coordinating Center: Jeffrey P. Krischer, Ph.D., PI[1,4,5,9,10], Rajesh Adusumali, Sarah Austin-Gonzalez, Maryouri Avendano, Sandra Baethke, Brant Burkhardt, Ph.D.[6], Martha Butterworth[2], Nicholas Cadigan, Joanna Clasen, Ph.D., Kevin Counts, Laura Gandolfo, Jennifer Garmeson, Veena Gowda, Shu Liu, Xiang Liu, Ph.D.[2,3,8,13], Kristian Lynch, Ph.D. [6,8], Jamie Malloy, Lazarus Mramba, Ph.D.[2], Cristina McCarthy[11], Hemang M. Parikh, Ph.D.[3,8], Cassandra Remedios, Chris Shaffer, Susan Smith[11], Noah Sulman, Ph.D., Roy Tamura, Ph.D.[1,2,11,12,13], Dena Tewey, Henri Thuma, Michael Toth, Ulla Uusitalo, Ph.D.[2], Kendra Vehik, Ph.D.[4,5,6,8,13], Ponni Vijayakandipan, Melissa Wroble, Jimin Yang, Ph.D., R.D.[2], Kenneth Young, Ph.D. *Past staff: Michael Abbondondolo, Lori Ballard, Rasheedah Brown, David Cuthbertson, Stephen Dankyi, Christopher Eberhard, Steven Fiske, David Hadley, Ph.D., Kathleen Heyman, Belinda Hsiao, Christina Karges, Francisco Perez Laras, Hye-Seung Lee, Ph.D., Qian Li, Ph.D., Colleen Maguire, Wendy McLeod, Aubrie Merrell, Steven Meulemans, Jose Moreno, Ryan Quigley, Laura Smith, Ph.D.* University of South Florida, Tampa, FL, USA.

Autoantibody Reference Laboratories: Liping Yu, M.D.^[5], Dongmei Miao, M.D.^, Kathleen Gillespie*[5], Kyla Chandler*, Olivia Pearce*, Sarah Stollery*, Elinor Balch*, Hanah Batholomew*, Zahra Hashmi*. ^Barbara Davis Center for Childhood Diabetes, University of Colorado Denver, *Bristol Medical School, University of Bristol, UK.

Dietary Biomarkers Laboratory: Iris Erlund, Ph.D.[2], Terhi Vihervaara Ph.D., Inga Liukko, Linda Patrikainen, Outi Ruotsalainen. Institute for Nutrition and Health Research, Institute for Nutrition and Health Research, Helsinki, Finland.

Genetics Laboratory: Stephen S. Rich, Ph.D.[3], Wei-Min Chen, Ph.D.[3], Suna Onengut-Gumuscu, Ph.D.[3], Emily Farber, Rebecca Roche Pickin, Ph.D., Jonathan Davis, Jordan Davis, Dan Gallo, Jessica Bonnie, Paul Campolieto. Center for Public Health Genomics, University of Virginia, Charlottesville, VA, USA.

HLA Reference Laboratory: William Hagopian[3], M.D., Ph.D., Jared Radtke. Pacific Northwest Research Institute, Seattle, WA, USA. (Previously Henry Erlich, Ph.D.[3], Steven J. Mack, Ph.D., Anna Lisa Fear. Center for Genetics, Children's Hospital Oakland Research Institute.)

Repository: Chris Deigan. NIDDK Biosample Repository at Fisher BioServices, Rockville, MD, USA. (Previously Ricky Schrock, Polina Malone, Sandra Ke, Niveen Mulholland, Ph.D.)

Project scientist: Beena Akolkar, Ph.D.[1,3,4,5,6,7,9,10]. National Institutes of Diabetes and Digestive and Kidney Diseases, Bethesda, MD, USA.

Other contributors: Thomas Briese, Ph.D.[6], Columbia University, New York, NY, USA. Todd Brusko, Ph.D.[5], University of Florida, Gainesville, FL, USA. Teresa Buckner, Ph.D.[2], University of Northern Colorado, Greeley, CO, USA. Suzanne Bennett Johnson, Ph.D.[8,11], Florida State University, Tallahassee, FL, USA. Eoin McKinney, Ph.D.[5], University of Cambridge, Cambridge, UK. Tomi Pastinen, M.D., Ph.D.[5,6], The Children's Mercy Hospital, Kansas City, MO, USA. Steffen Ullitz Thorsen, M.D., Ph.D.[2], Department of Clinical Immunology, University of Copenhagen, Copenhagen, Denmark, and Department of Pediatrics and Adolescents, Copenhagen University Hospital, Herlev, Denmark. Eric Triplett, Ph.D.[6], University of Florida, Gainesville, FL, USA.

Committees:

[1]Ancillary Studies, [2]Diet, [3]Genetics, [4]Human Subjects/Publicity/Publications, [5]Immune Markers, [6]Infectious Agents, [7]Laboratory Implementation, [8]Psychosocial, [9]Quality Assurance, [10]Steering, [11]Study Coordinators, [12]Celiac Disease, [13]Clinical Implementation.
